# Comparative Bilateral Measurements of Vastus Lateralis Muscle Oxygen Desaturation Kinetics during 30 S Sprint Cycling Exercise: Effects of Age and Performance

**DOI:** 10.3390/jfmk9020104

**Published:** 2024-06-13

**Authors:** Karmen Reinpõld, Indrek Rannama, Kristjan Port

**Affiliations:** School of Natural Sciences and Health, University of Tallinn, 10120 Tallinn, Estonia; rannama@tlu.ee

**Keywords:** NIRS, Moxy Monitor, phosphocreatine, anaerobic capacity

## Abstract

The study assessed vastus lateralis oxygen desaturation kinetics (SmO_2_) in 32 male cyclists (16 Seniors, 16 Juniors) during a 30 s sprint, examining effects of age and performance. An incremental test was used to determine ventilatory thresholds (VT1, VT2) and maximal oxygen uptake (VO_2kg_), followed by a sprint test to evaluate anaerobic performance. Cyclists’ performance phenotype was determined as the ratio of power at VT2 to 5 s peak sprint power. Juniors exhibited sprinter-like traits, excelling in all functional tests except for lactate levels post-sprint. SmO_2_ data showed no age-related or bilateral differences across participants. The combined mean response time (MRT) revealed stronger bilateral goodness of fit (R^2^ = 0.64) than individual time delay (TD) and time constant (τ). Higher VO_2kg_ at VT2, peak power, and maximal uptake were linked to longer TD, while shorter TD correlated with higher lactate production and increased fatigue. Bilaterally averaged SmO_2_ kinetics distinguished between sprint and endurance athletes, indicating the potential to reflect the alactic anaerobic system’s capacity and depletion. Age did not affect desaturation rates, but younger cyclists showed greater response amplitude, attributed to a higher initial baseline rather than maximal desaturation at the end of the exercise.

## 1. Introduction

Cycling, typically lasting several hours, is classified as an endurance sport, though it maintains a complex relationship with maximal sprint efforts. Depending on the course profile, the race often includes and concludes with a maximal sprint finish, performed under conditions of high aerobic load, accumulated fatigue, and relative energy deficiency, leading to decreased overall efficiency [1]. Therefore, despite relatively short maximal efforts, the contribution of aerobic, alongside with anaerobic, energy provision is considerable [2].

Currently, it is common and straightforward for coaches to observe and analyse training data, including power output and heart rate, to assess expected developments in aerobic performance, such as anaerobic threshold power or the capacity for maximal oxygen uptake [3]. However, evaluating and comprehending the impact and changes in aerobic power during short-term efforts is not straightforward [2]. Data collected during sprint tests could be highly valuable if it offers insights into the role of both aerobic and anaerobic energy pathways, their relative proportions, and how these evolve over time.

Lately, there has been a rise in popularity of Near-infrared Spectroscopy (NIRS) application during the training processes, evidenced by the increasing number of related scientific publications [4]. Most daily practices and research literature focus on monitoring and evaluating long, steady-state aerobic efforts [5,6]. Nevertheless, accumulating knowledge is beginning to shed light on how NIRS offers insights into the rapid activation of various energy systems during maximal effort [7]. Although, this challenge cannot be resolved overnight, but it provides a compelling reason to add data collected using more recent technologies.

NIRS studies focusing on short-duration exercises have often been conducted with a heterogeneous group of recreational athletes [7]. However, the same experimental details are particularly important to coaching professional athletes. There have been encouraging studies by Dunst et al. [8] involving elite-level track sprint cyclists. Furthermore, it would be insightful to investigate whether and how an athlete’s phenotype (endurance-oriented or sprinter-like), along with their training history, experience, and age, influences oxygen kinetics at the muscle during maximal effort. Initially, well-trained Seniors have extensive aerobic training exposure compared to younger, high-performing athletes with less training experience [9]. As such, there may be a distinctive adaptation on a local muscle level with years of specific endurance work, which could contribute to potential variation [10,11]. It will be advantageous to understand whether there are differences in oxygen dissociation kinetics from carrier proteins when comparing athletes with a sprinter-like profile to those with an endurance-like profile, and if this information reveals any advantages [12]? Our recent study has demonstrated the cyclist’s phenotype-related associations with oxygen saturation dynamics during aerobic cycling in incremental exercise conditions [9], but there is no information about the athlete’s typology or performance-related effects on deoxygenation dynamics in working muscle during maximal sprinting exercises.

When measuring local oxygen utilisation with the NIRS system, it is often used only on one leg—either on the dominant or non-dominant leg [13,14]. Recent studies [9,15] have introduced new findings regarding the importance of bilateral signal recording for a comprehensive understanding of the processes contributing to power output. Evidence suggests that oxygen saturation signals from the primary power-generating muscles in the contralateral legs do not adjust in bilateral synchrony during steady-state aerobic and incremental cycling efforts [9,15], but no pieces of evidence are presented in the scientific literature about bilateral deoxygenation processes during maximal sprinting exercises.

Key agonistic muscle groups are expected to achieve consistently coordinated peak work output with no systematic bilateral differences at maximum effort in sprint cycling [16]. Consequently, the knee extensors’ muscle activity has demonstrated no between-body-sides differences during 30 s sprint cycling for trained cyclists [17], and, therefore, the kinetics of muscle oxygen saturation among those muscles should show a high bilateral correlation, but knowledge is lacking regarding bilaterally measured muscle oxygen saturation (SmO_2_) kinetics during fatiguing sprint cycling exercises. 

Coaches are eager to transition from laboratory testing to field testing to increase mobility [18,19]. Aerobic performance is assessed daily. Still, this is only half the picture of understanding the athlete’s potential and limiting aspects. Hence, a more refined comprehension of NIRS signal conveying information during short, minimally taxing, and repeatable sprints for evaluating the contribution of the anaerobic energy systems could serve as an inexpensive, timesaving (compared to laboratory testing), and informative field test. Two of our research group’s latest publications [9,15] have focused on different aspects of bilaterally measured oxygen desaturation dynamics in the trained cyclist population during intensive aerobic cycling exercises where muscle O_2_ consumption is firmly delivery dependent [20,21]. However, the current study aims to fill the gap in knowledge about the informativity of oxygen saturation kinetics during maximal sprinting, when O_2_ saturation should mainly not be delivery dependent [6,22].

The aim of the current study was to evaluate the agreement between bilaterally measured characteristics of vastus lateralis oxygen desaturation kinetics during a 30 s sprint cycling exercise in well-trained cyclists of different ages, and therefore long term training exposure, and analyse the effect of cyclists’ age and performance on characteristics of muscle oxygen desaturation kinetics according to NIRS.

## 2. Materials and Methods

### 2.1. Participants 

This study involved 16 experienced male cyclists aged 37–52 (grouped as Seniors) and 16 Junior and U23 class (grouped as Juniors) national- and international-level cyclists (detailed description in Table 1). The Senior cyclists have been continuously training for over a decade, with more than 5000 km per year, although none have cycled professionally. They have been athletically active since childhood but turned to performance-oriented cycling in later years. The Junior cyclists have been training for 4 to 8 years, covering over 10,000 km annually. Cyclists with insufficient training histories or health issues were not included. Participants were instructed to follow their usual balanced diet, refrain from intense physical activity for 48 h before the test, and not consume caffeine on test day. All participants were accustomed to laboratory tests. 

### 2.2. Study Design

The study utilised a cross-sectional design to evaluate cyclists’ oxygen saturation (SmO_2_) dynamics in the vastus lateralis muscle using NIRS. Each cyclist’s testing was completed in one day during the early competitive season, between February and March. The participants performed an incremental cycling exercise followed after rest by a 30 s sprint test using their own bicycles to assess cycling performance and physiological response to exertion.

### 2.3. Procedures and Instrumentations

Upon arriving at the laboratory, participants completed questionnaires detailing their training history, including the number of training years over 5000 km annually (verified with electronical training diaries), the total distance cycled in the previous year, and their training objectives. The anthropometric data, such as height and body mass, were measured using a Seca mBCA 514/515 body composition analyser (Medical Measurement Systems and Scales, Hamburg, Germany). Skinfold thickness was measured at the NIRS sensor site using a Baseline Skinfold Caliper (Fabrication Enterprises, Inc., New York, NY, USA). The cyclists’ leg dominance was evaluated by ball-kicking preference, and all cyclists, except for one Junior, were right-legged. 

All performance tests were conducted on the athletes’ road bikes mounted on the Cyclus2 cycle ergometer (RBM Elektronik-Automation, Leipzig, Germany) [23]. The experimental procedure was divided into two segments. The initial phase commenced with an incremental cycling test designed to evaluate the aerobic performance capabilities of the cyclists. The incremental test protocol started with a 7 min warm-up at 90 W; initial power was set at 100 W, and workload increased by 30 W every three minutes (target cadence of 90–100 rpm) until athletes stopped because of fatiguing or pedalling cadence dropped below 70 rpm. During the incremental test, a breath-by-breath gas analyser captured the respiratory and pulmonary gas exchange variables (Quark PFTergo, Cosmed, Rome, Italy). The gas analyser was calibrated before each test using reference gases (5% CO_2_; 16% O_2_) and a known volume syringe (3 L), following the manufacturer’s instructions. The Cyclus2 ergometer was operated by the Cosmed OMNIA 2.1 (Cosmed, Rome, Italy) gas analysis software during incremental test, and the same software was used for capturing metabolic measures and evaluating aerobic performance abilities.

The second phase of the experiment consisted of a 30 s all-out sprint test. At least a 20 min active recovery period was included between the experimental phases, consisting of a 10 min ride at 100 W and at least a 10 min ride at 80% of the power output at the first ventilatory threshold (VT1). When capillary blood lactate level was lowered to less than two mmol/L, then the 30 s sprint cycling test was conducted in the seated position, with hands-on drops in isokinetic mode with a fixed cadence of 110 rpm, to determine the athlete’s anaerobic performance abilities and to measure oxygen saturation kinetics. The dynamics of power and cadence during sprint cycling were captured by Cyclus2 head unit software (Release 5.0) at a 10 Hz sampling rate. After the end of sprint cycling, the athletes stopped pedalling and sat still until their lactate level started to lower systematically. Cyclist lactate level was measured with Diaglobal DP110 Lactate Photometer plus (Diaglobal GmbH, Berlin, Germany) from the finger after the incremental cycling exercise to monitor lactate dynamics. The lactate concentration (LA_pre_) was measured just before the sprint cycling test and every minute after sprinting until peak value (LA_max_) was achieved to evaluate the lactate accumulation rate. 

Oxygen saturation (SmO_2_) in participants’ vastus lateralis muscles was recorded bilaterally using the Moxy Monitor (Fortiori Design LLC, Hutchinson, MN, USA) NIRS probes, established as valid and reliable during aerobic conditions and arterial occlusion [4,22,24]. The Moxy Monitor utilises a Near-Infrared light source that emits light at four wavelengths ranging from 630 to 850 nm. The light is detected by two sensors located 12.5 mm and 25 mm away from the emitter. This device operates with an output sampling rate of 1 Hz. For signal processing, it employs the Monte Carlo model, which is elaborated upon further in another source [14]. The device was affixed approximately two-thirds of the distance between the anterior superior iliac spine and the lateral side of the patella [4] on both legs and held in place with medical tape. Data were collected at a frequency of 1 Hz, transmitted via ANT+ to a Garmin Edge 520 (Garmin, Olathe, KS, USA) head unit, and synchronised with a Favero Assioma Duo power meter (Favero Electronics, srl., Arcade, TV, Italy) for bilaterally measured power data.

### 2.4. Computations and Measurements

The cyclists’ adipose tissue thickness (ATT) was computed as half of the measured skinfold thickness [25] to evaluate the suitability of participants to meet the criteria to use the Moxy Monitor device [14]. Analyses of the ventilatory threshold were conducted using the Cosmed OMNIA 2.1 software (Cosmed, Rome, Italy) to assess aerobic performance capabilities. The first ventilatory threshold (VT1) was determined as the first breakpoint of the V̇CO_2_/V̇O_2_ vs. power. The second ventilatory threshold (VT2) was identified based on (a) a second slope increase on the curve between minute ventilation (VE) and power, (b) a second increase in ventilatory equivalent for oxygen and ventilatory equivalent for carbon dioxide, and (c) a decrease in end-tidal partial pressure of carbon dioxide, as outlined by Wasserman [26]. The highest 30 s mean value of V̇O_2_ was recorded as the V̇O_2max_. The average 30 s V̇O_2_ values around the time point of ventilator thresholds were used as V̇O_2_ characteristics at VT1 (V̇O_2_ @VT1) and V̇O_2_ (V̇O_2_ @VT2). Power (P [W]) values for Peak Power (PPW), VT1 (P@VT1), and VT2 (P@VT2) were calculated proportionally using the time when the respective criteria were achieved:

(1) $$P\left[W\right]=P_{last\;completed\;workload}\left[W\right]+30\left[W\right]\times \frac{t_{uncompleted\;workload}\left[s\right]}{180\left[s\right]}$$

The relative (normalised with body mass) threshold and peak values of P [W/kg] and V̇O_2_ [mL/kg/min] were used as indicators of the athletes’ aerobic performance. The anaerobic power and capacity were described by relative power [W/kg] of the highest 5 s (Pmax5s) and average 30 s sprint power (Pmax30s) captured by Cyclus2 ergometer software (version 5.0) during the sprint test and also the lowest 5 s power (Pmin5s) value at the end of the sprint was measured. The explosiveness of the athlete was described by time to achieve the peak power (t@Pmax [s]) and rate of power development (RPD) for achieving 90% of Pmax from the pre-test baseline (P_BL_) level (~70 W):

(2) $$RPD[W/kg/s]=\frac{90\%Pmax-P_{BL}}{t@90\%Pmax}$$

The fatigue development during the sprint was evaluated by relative power drop (PD) expressed as the fatigue index (FI):

(3) PD[W/kg]=Pmax5s−Pmin5s

(4) $$FI\left[\%\right]=100\times \frac{Pmax5s-Pmin5s}{Pmax5s}$$

The cyclists’ glycolytic power during the sprint was described by the Lactate accumulation rate (V_LAmax_):

(5) $$V_{LAmax}[mMol/L/s]=\frac{{LA}_{max}-{LA}_{pre}}{30-t@Pmax}$$

For the current study, participants were further grouped according to relative balance between sprinting and aerobic endurance capabilities, quantitatively expressed as the ratio between Pmax5s and P@VT2 [au] and given as a corresponding “sprinter-like” phenotype [9].

Bilateral power data measured by Favero Assioma Duo power meter (Favero Electronics, srl., Arcade, TV, Italy) at 1 Hz was used to evaluate Pmax5s, Pmax30s, Pmin5s, PD, and FI values for ND and DO leg according to formulae presented previously.

The VL muscle O_2_ saturation (SmO_2_) signal was captured at 1 Hz signal in the original scale from 0 to 100%, and it was pre-processed with algorithms provided by the manufacturer and described in detail by Feldmann, Schmitz, and Erlacher [14]. The time axis of SmO_2_ data was aligned such that time “0” represented the start of sprinting, and 10 s data before the sprint started during cycling at 70 W with a cadence of 100 rpm was used to measure baseline value. Additionally, to ND and DO SmO_2_ signals also, the average (Avr) signal of both legs was computed because aggregated SmO_2_ values of two body sides have demonstrated higher agreement with signals of systemic metabolic reactions [9,15]. For correlational analyses between SmO_2_ kinetics and performance measures, only AvrSmO_2_ measures were incorporated.

**Figure 1:**
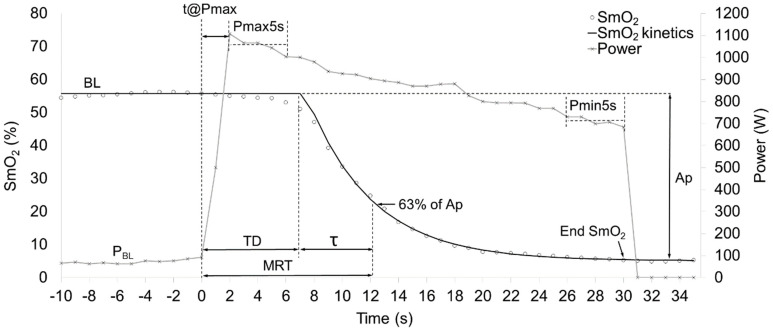
Illustration of 30 s sprint effort and measured and calculated parameters. Baseline (BL), time to achieve peak power (t@Pmax), maximal average 5 s power (Pmax5s), minimal average 5 s power (Pmin5s), baseline power (P**_BL_**), time delay (TD), time constant (τ), mean response time (MRT), minimal oxygen saturation value (End SmO_2_), amplitude (A_p_), oxygen saturation (SmO_2_).

The SmO_2_ kinetics of all three signals were modelled with R language-based open-source software VO2FITTING ver. 0.46 [27] with monoexponential regression analysis with Heaviside (H) step function:

(6) $${SmO}_{2}\left(t\right)=BL-H\left(t-TD\right)\times A_{p}\left(1-e^{-\frac{t-TD}{\tau }}\right);\;H\left(t\right)=\left\{\begin{array}{c} 0,\;t<0\\ 1,\;t\geq 0 \end{array}\right.$$

where t represents the time; BL is the average 10 s baseline value before sprint start; A_p_, TD_p_ and TD (s), and τ are the response amplitudes, the corresponding time delays, and time constant of the function, respectively; and H represents the Heaviside step function [27]. Additionally, the mean response time (MRT) was computed as a sum of TD and τ and the End SmO_2_ value was computed by equation [6] at the end of sprinting (t = 30) to describe the lowest SmO_2_ value achieved during sprinting (Figure 1). The goodness of the model’s fit to actual data was described by the standard error of the regression (SE_regr_).

### 2.5. Statistical Analysis

The descriptive statistics for measured characteristics are presented as mean and standard deviation (SD), and the relative variance of measurements is described by the coefficient of variation (CoV). The assumption for data normality was controlled with the Shapiro–Wilk normality test, and for Age Group comparisons, the equality of variances was tested with Levene’s test. The differences between Age Group characteristics were controlled with Student’s *t*-tests for independent samples or with the Mann–Whitney test in cases where assumptions for parametric tests were not met. The Student’s *t*-test for paired samples was used to control the differences between ND and DO leg variables. The ANOVA for repeated measures was used to control combined effects of Age Group (between-subject factor) and leg dominance (repeated measure) to SmO_2_ kinetics or 30 s power dynamics values. The Bonferroni post hoc test was applied if the general ANOVA model indicated significant differences.

The Bland Altman analysis evaluated the agreement level between ND and DO SmO_2_ kinetics characteristics on the whole sample and for separate age groups with upper and lower limits of agreement (LOA) at 1.96 SD level. A simple regression analysis and plots were conducted to evaluate agreement in covariation between ND and DO SmO_2_ kinetics values, and the coefficient of determination (R^2^) was used to measure the strength of associations. 

Correlation and partial correlation analyses were performed to analyse the associations between cycling-specific performance characteristics and SmO_2_ kinetics variables. The Shapiro pairwise normality test was used to control the assumption for parametric correlation analysis. The nonparametric Spearman’s correlation was used if the assumption was not met. For the interpretation of correlation coefficients, the threshold levels 0.1, 0.3, 0.5, 0.7, and 0.9 for small, moderate, large, very large, and extremely large were used [28].

Statistical analysis was performed using JASP 0.18.3 software (JASP Team, 2024). The level of statistical significance was set at *p* < 0.05. Additionally, the effect size of Cohen’s d > 0.2 was used to confirm the statistical difference in the *t*-test results. Interpretation of the effect size is as follows: 0.2 = small effect; 0.5 = moderate effect; 0.8 = large effect [28].

## 3. Results

### 3.1. Performance Characteristics

Table 2 presents the characteristics of aerobic and anaerobic performance per body mass. Except for lactate production capabilities, all the parameters were significantly (*p* < 0.05; d > 1) higher for Junior athletes than Seniors. Juniors displayed a tendency towards a sprinter-like typology, yet they also experienced fatigue at an accelerated rate during sprinting activities.

**Figure 2:**
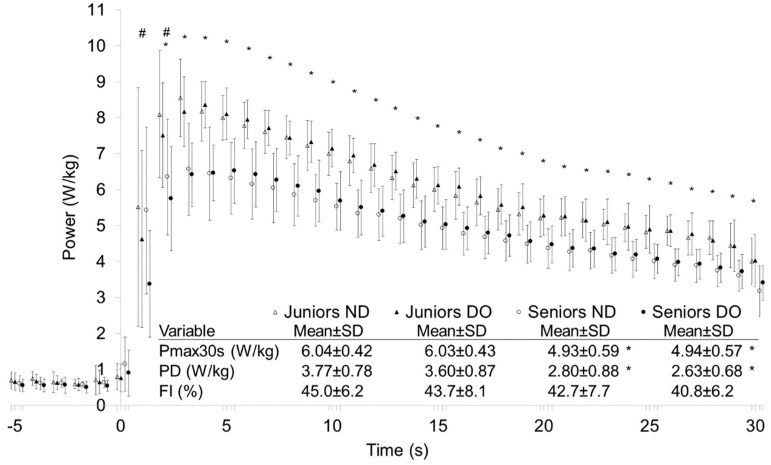
Bilateral power dynamics of non-dominant (ND) (open symbols) and dominant (DO) (closed symbols) leg during sprinting in Juniors (triangles) and Seniors (circles) age groups. Maximal average 30 s power (Pmax30s); power drop (PD); fatigue index (FI). *—significant difference between Juniors and Seniors; (*p* < 0.05; d > 0.2); #—significant difference between DO and ND leg; (*p* < 0.05; d > 0.2).

Figure 2 presents bilaterally measured power dynamics of Juniors and Seniors during 30 s sprints with 1 s time intervals. The average power of Juniors was significantly (*p* < 0.05) higher during the whole test for both legs, except during the first second. There was a significant (*p* < 0.05) bias for higher power production of the ND leg during the first 2 s for both age groups, but no bilateral differences were presented after that, as well as between bilateral values of 30 s average power, PD, and FI in the whole sample and age groups.

### 3.2. SmO_2_ Kinetics during 30 s Sprint Effort and Agreement between Bilateral Values

SmO_2_ kinetics during sprinting followed good monoexponential fit with TD (R^2^ > 0.98 for all cases) and modelled characteristics for two age groups are presented in Table 3. There were no between-group differences in time-related measurements. Only the baseline values for Juniors were significantly (*p* < 0.05) higher; therefore, the SmO_2_ response amplitude values were also greater. There were no bilateral differences (*p* > 0.05) in any of the measured parameters in either Age Group and consequently in the total participant pool.

**Figure 3:**
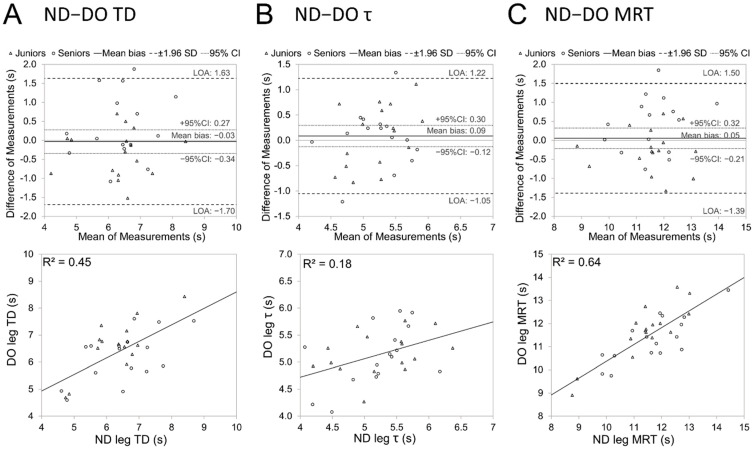
Bilateral agreement between time variables of SmO_2_ kinetics during 30 s sprint cycling. Non-dominant leg (ND), dominant leg (DO), time delay (TD) (**A**), time constant (τ) (**B**), mean response time (MRT) (**C**).

Figure 3 demonstrates Bland Altman and regression plots for the size of bilateral agreement between time variables of SmO_2_ kinetics during 30 s sprint cycling. Shared MRT measure demonstrated stronger bilateral goodness of fit (R^2^ = 0.64) compared to individual TD and τ parameters.

**Figure 4:**
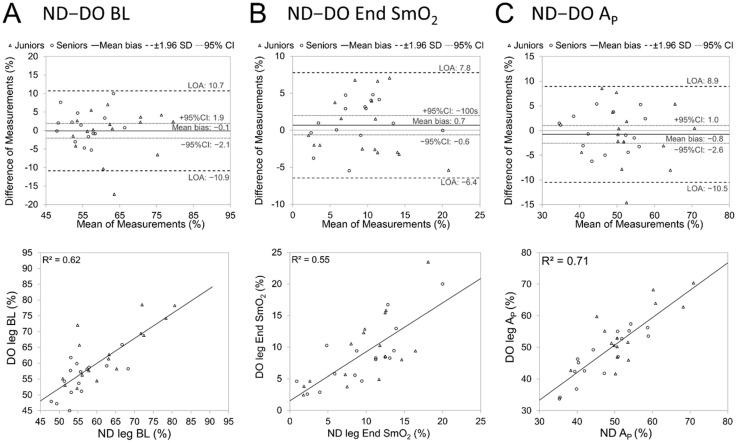
Bilateral agreement between SmO_2_ amplitude variables of SmO_2_ kinetics during 30 s sprint cycling. Non-dominant leg (ND), dominant leg (DO), baseline (BL) (**A**), minimal oxygen saturation value (End SmO_2_) (**B**), amplitude (A_p_) (**C**).

Bilateral agreements between SmO_2_ amplitude variables of SmO_2_ kinetics are illustrated in Figure 4. The response amplitude showed a higher goodness of fit (R^2^ = 0.71) between legs than the same result for the Baseline and End SmO_2_.

### 3.3. Associations between SmO_2_ Kinetics and Cycling Performance

Table 4 presents the partial correlation analysis results between cyclists’ performance characteristics and SmO_2_ kinetics parameters measured during a 30 s sprint effort across the entire participant pool. Age Group was used as a controlling variable to eliminate the moderating effect of performance-level differences between age groups.

**Figure 5:**
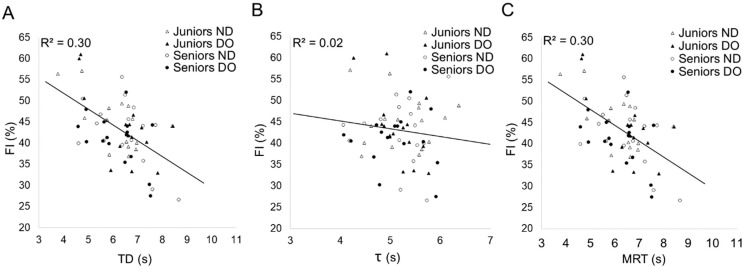
Relationship of fatigue measures and time variables of SmO_2_ kinetics across all participants, considering both dominant (DO) and non-dominant (ND) legs: Fatigue index (FI) and time delay (TD) (**A**), time constant (τ) (**B**), mean response time (MRT) (**C**).

The correlation analysis (Table 4) indicates that individuals with higher relative VO_2kg_ levels at VT2, peak power and maximal oxygen uptake—therefore, more endurance-type athletes—tend to have longer TD and vice versa. Also, the shorter TD was associated with a higher lactate production rate and greater fatigue parameters. As expected, similar trends are observable in TD incorporating MRT—athletes with better aerobic fitness exhibit longer MRT compared to those with a more anaerobic training background, who also experience more pronounced fatigue. At the same time, τ had no independent relationship with the measures of performance. The same pattern between the fatigue index and time variables can be observed in Figure 5. There were no correlations between SmO_2_ saturation parameters and performance characteristics.

## 4. Discussion

A primary objective of this study was to assess the vastus lateralis muscle oxygen desaturation kinetics among well-trained Senior and Junior road cyclists and analyse the associations between cycling-performance-related abilities and characteristics of SmO_2_ kinetics.

Overall, the desaturation kinetics of all cyclists demonstrated a similar delayed phase followed by monoexponential decay in the SmO_2_ signal and culminating in a plateau towards the end of the effort. Similar findings were also observed in the study of Dunst et al. [8] during a 60 s maximal sprint effort among elite-level track sprinters. In the aforementioned study, there is limited detailed discussion on the kinetics of SmO_2_. For instance, although TD was used in calculations, its functional significance was not addressed. In the current study, TD is found to have a high correlation with individual performance characteristics. As expected, this was clearly observed in the MRP, which represents the sum of the time delay (TD) and the desaturation decay time constant. Some authors have suggested that the entire duration of TD may not be related to physiological processes. It is speculated that an unknown portion of TD could be systematically biased due to signal computation and processing by the Moxy Monitor [29]. 

Direct invasive measurements of muscle O_2_ consumption have demonstrated time differences of up to 15 s between the start of workload and rise in arterial-venous O_2_ difference in working muscle during moderate-intensity cycling for untrained participants [20]. The overlap of this physiological event in time with TD should not be considered insignificant. The current study found slightly over the 6 s SmO_2_ TD values for both age groups measured during a maximal sprint cycling exercise, being in good agreement with a previous study transitioning from baseline (120–150 W) to heavy (VT2) intensity [15]. O_2_ desaturation TD values for young trained cyclists have been reported to average 8.2 ± 1.5 s at workload transition from the low baseline to moderate intensity and 6.1 ± 1.8 s at workload transition to heavy intensity [30]. For higher workloads (above VT2) under rapidly rising ATP demands, the TD may correlate with the capacity for alactic anaerobic ATP resynthesis [22]. Expanding on this idea, the combined measure of MRT aligns well with the known capacity of the phosphocreatine energy store, which can be largely depleted within 10–15 s of all-out exercise [21]. This notion is supported by the findings of the present study, where shorter TDs and MRTs were linked to a more sprinter-like phenotype (i.e., explosive power generation), while a longer TD was associated with more pronounced aerobic abilities. Furthermore, shorter TDs showed association with faster fatigue rates and higher V_LAmax_ measures. 

Unlike TD, the τ seems sensitive to rate of work above VT2 intensity. In our previously mentioned study with a similar sample of senior cyclists [15], the average τ was 13.7 ± 3.4 s when transitioning from a lower rate to the VT2 level. However, in the current study, the τ was more than twice as short during sprint exercise. Compared to elite track sprinters in the study by Dunst et al. [8], our endurance-type road cyclist participants exhibited a more extended time constant, approximately 3 s versus 5 s, calculated by comparable methods from signals captured by the same instrument. Sprinters appear to exhibit quicker oxygen desaturation kinetics, which could be attributed to the relatively faster utilisation of oxygen. Since there was a significant partial correlation with the end effort Pmin5s, τ might encompass insights into the rate at which energy-rich phosphates are depleted under the NIRS detector area, also concluded by Dunst et al. [8]. The rate of O_2_ dissociation from carrier proteins haemoglobin and myoglobin under the probe may indicate the contribution of glycolysis, as evidenced by the negative correlations between V_LAmax_ and MRT in the current study. This phenomenon is further supported by the comparison of slower SmO_2_ kinetics in endurance-adapted road cyclists, who also have lower V_LAmax_ values of 0.43 mmol/L/s, compared to 0.95 mmol/L/s in elite track sprinters [8].

Our study also aimed to analyse the effect of cyclists’ age on muscle oxygen desaturation kinetics. Despite the differing aerobic and sprinting abilities between Junior and Senior participants in the current study, there were no inter-group differences in the informationally monotonous time domain measures of SmO_2_ kinetics. However, the response amplitude (A_p_) in the Juniors (53.6%) group was significantly higher than for Seniors (46.7%). The larger A_p_ for Juniors was related to higher BL SmO_2_ values before sprinting, while the end-of-exhaustion SmO_2_ did not differ between groups. This may be explained by the relatively lower baseline power (70 W) for Juniors, given their higher aerobic capabilities. Alternatively, it is more likely attributable to the sprinter-like characteristics of the Junior cyclists. It has been found that sprinter-type cyclists exhibit a higher proportion of less oxidative, glycolytic type II muscle fibres in the vastus lateralis muscle [31], likely located in more superficial layers [31,32], accessible to the NIRS probe. This assumption is supported by even higher BL SmO_2_ (75.13 ± 8.60%) and A_p_ (65.94 ± 9.36%) values for elite track sprinters during a 60 s sprint effort [8] than demonstrated in the present study. The End SmO_2_ values of elite track sprinters in Dunsts et al.’s [8] study were on average slightly below 10%, similar to both age groups in the current study. Similarly, a previous study with comparable age groups found near 10% minimum SmO_2_ values at the end of a stepwise cycling test to exhaustion [9]. This further suggests that a 30 s sprint exercise can achieve similar O_2_ desaturation levels as those reached at the end of longer, more aerobically demanding, exhaustive exercise. This knowledge may be helpful for situations where SmO_2_ minimal value for normalisation of physiological amplitude is needed.

The study also aimed to investigate whether there is significant variation in oxygen desaturation kinetics between legs during intense physical effort. It was hypothesised that, unlike lower exercise intensities—where relatively low contralateral agreement and some bilateral compensatory or sharing effects have been observed [9,15]—during sprinting, when both legs must exert maximum effort, the contralateral agreement between SmO_2_ variables would be more consistent. The current study did not show a significant difference between the characteristics of the dominant and non-dominant legs. The Bland Altman and regression analyses demonstrated poor within- and between-individual bilateral agreement in oxygen desaturation TD and τ measures, showing improvement for MRP, combining information from both TD and τ domains. Those findings are at least partly related to the methodological limitations of the current study: the low sampling rate (1 Hz) of the SmO_2_ signal can cause up to 1 s random error in timing values, depending on how precisely the actual start of sprinting action by cyclists was aligned with sampling timing. Another issue comes from the bilaterally equal but out-of-phase nature of cycling movement—the time lag between the initiating force momentum on a pedal and contralateral leg is at least 0.27 s (110 rpm) during the pedalling cycle. That may also cause time delays in metabolic reactions. Study participants demonstrated a significant (*p* < 0.05) inclination towards greater power output from the ND leg during the first 2 s of sprinting in both age groups, and this might have influenced the bilateral differences in time variables of SmO_2_ kinetics as well. The relatively higher agreement between contralateral MRT variables, compared to TD and τ, also indicates the aforementioned methodological biases, demonstrating that the summation of TD and τ increases the bilateral accordance. At the same time, the previously publicised results of bilateral SmO_2_ fast kinetics in the heavy intensity domain have demonstrated even lower group-level agreement between contralateral legs [15], expressed as more than twofold smaller common variability between all contralateral time variables, compared to the results of the present study.

The bilateral SmO_2_ values (BL, Ap, and End SmO_2_) showed higher group-level agreement than TD and τ, but were similar to MRT. This level of agreement is consistent with reported breakpoints of contralateral SmO_2_ signals during incremental exercise [9]. The Bland Altman analysis did not demonstrate differences in any of SmO_2_ amplitude values between working legs, and intraindividual differences as limits of agreement (LOA) were within the range of −11 to 11% for BL, Ap, and even narrower values for End SmO_2_ measure. Similar or wider LOA are reported for day-to-day agreement between minimum and maximum SmO_2_ measures for the same VL muscle site in the Moxy Monitor validation study [14], indicating that during sprinting, the contralateral agreement is comparable to or even higher than the between-day agreement for the same muscle site. The bilateral agreement between SmO_2_ amplitude characteristics may be influenced by morphological asymmetries like ATT [25] and sensor placement [33]. 

The following methodological limitations of this study must be considered when interpreting the presented results. As stated above, the low sampling rate of SmO_2_ signal may be contributing to up to 1 s random error in time-related characteristics. Due to the study design, the sprint was conducted after the incremental exercise; therefore, the possible effect of fatigue may influence the metabolic processes and mechanical power generation during the 30 s sprint effort. Also, the two age groups in our study differed not only in chronological age but also in their training backgrounds and performance levels. This overlap complicates the ability to distinctly attribute the observed discrepancies in measured values to either age differences or variations in training status.

Our study suggests the speed of SmO_2_ kinetics is indicative of the capacity and depletion rate of the alactic anaerobic energy system. For the practical application, it is not reasonably easy to compute the kinetics in everyday practice. At the same time, MRT carries important information; therefore, it would be feasible to measure the time from the start of the power generation (SmO_2_ baseline) until the time when it reaches 63% of response amplitude (minimum value of SmO_2_). The average power value during the MRT may reflect the anaerobic alactic capacity, and this measure can be a possible marker to evaluate anaerobic alactic abilities during training processes. Although, this has to be confirmed in future studies.

## 5. Conclusions

The present study demonstrated that during short-term, maximal-effort sprint cycling, the vastus lateralis muscle area under the NIRS sensor experienced rapid oxygen desaturation from carrier proteins, following a notable initial delay.

Despite the relatively low bilateral consistency among most time-based measures of SmO_2_ kinetics, the time domain measures of bilaterally averaged SmO_2_ signal kinetics effectively differentiated between sprint- and endurance-type athletes and suggested potential for indicating the capacity and depletion rate of the alactic anaerobic energy system.

The differences in cyclists’ ages did not manifest in the rate of oxygen desaturation kinetics; however, younger cyclists exhibited a greater response amplitude, likely related to a higher initial functional baseline rather than the maximal desaturation at the end of the exercise.

## Figures and Tables

**Table 1:** The anthropometric and training-history-related characteristics of study participants grouped as Junior and Senior cyclists.

Variable	Juniors				Seniors				
	N	Mean	SD	CoV	N	Mean	SD	CoV	
Age (y)	16	18.2	1.6	0.09	16	43.3	4.9	0.11	*
Height (m)	16	1.858	0.047	0.03	16	1.830	0.055	0.03	
Body Mass (kg)	16	73.7	4.6	0.06	16	82.0	6.6	0.08	*
BMI (body mass index)	16	21.4	1.5	0.07	16	24.5	1.7	0.07	*
ATT (adipose tissue thickness) (mm)	16	3.8	1.2	0.33	16	6.5	1.9	0.29	*
Cycling training status (years)	16	5.4	1.1	0.20	16	17.4	5.4	0.31	*
Seasonal cycling distance (km)	16	17,063	2839	0.17	16	10,488	3072	0.29	*

*—significant difference between Juniors and Seniors (*p* < 0.05; d > 0.2).

**Table 2:** Characteristics of aerobic and anaerobic performance of Junior and Senior cyclists. Power (P), 1st ventilatory threshold (VT1), 2nd ventilatory threshold (VT2), peak power (PP), oxygen uptake (VO_2_), rate of power development (RPD), time (t), power drop (PD), fatigue index (FI), lactate accumulation rate (VLa_max_).

Variable	Juniors				Seniors				*p*	Effect Size
	N	Mean	SD	CoV	N	Mean	SD	CoV		
P@VT1 (W/kg)	16	3.25	0.25	0.08	16	2.87	0.41	0.14	0.003	1.14
P@VT2 (W/kg)	16	4.32	0.29	0.07	16	3.65	0.39	0.11	<0.001	1.94
PPW (W/kg)	16	5.31	0.33	0.06	16	4.44	0.47	0.11	<0.001	2.17
VO_2_@VT1 (mL/min/kg)	16	44.8	3.9	0.09	16	42.2	4.8	0.11	0.095	0.61
VO_2_@VT2 (mL/min/kg)	16	58.4	3.5	0.06	16	52.8	5.5	0.10	0.002	1.21
VO_2max_ (mL/min/kg)	16	67.6	4.3	0.06	16	60.7	6.4	0.11	0.001	1.28
RPD (W/s/kg)	16	15.2	7.6	0.50	16	7.8	3.6	0.47	0.001	1.26
t@Pmax	16	2.00	1.09	0.55	16	2.99	1.40	0.47	0.033	−0.79
Pmax1s (W/kg)	16	17.7	1.8	0.10	16	13.5	1.8	0.13	<0.001	2.32
Pmax5s (W/kg)	16	16.8	1.6	0.09	16	13.0	1.8	0.14	<0.001	2.29
Pmax30s (W/kg)	16	12.2	0.9	0.07	16	9.9	1.1	0.11	<0.001	2.39
Pmin5s (W/kg)	16	9.3	0.9	0.10	16	7.9	0.7	0.09	<0.001	1.78
PD(W/kg)	16	7.5	1.7	0.22	16	5.1	1.4	0.28	<0.001	1.51
FI (%)	16	44.2	6.3	0.14	16	38.9	6.6	0.17	0.025	0.83
V_LAmax_ (mmol/L/s)	16	0.43	0.08	0.18	16	0.43	0.10	0.24	0.975	0.01
Phenotype (a.u.)	16	3.90	0.41	0.11	16	3.57	0.43	0.12	0.032	0.79

**Table 3:** SmO_2_ kinetics parameters measured during 30 s sprint effort for the non-dominant (ND) and dominant (DO) leg and averaged (Avr) signal. Time delay (TD), time constant (τ), mean response time (MRT), baseline (BL), minimal oxygen saturation value (End SmO_2_), amplitude (A_p_), standard error of regression (SE_regr_).

Variable	Group	N	ND			DO			Avr		
			Mean	SD		Mean	SD		Mean	SD	
TD (s)	Juniors	16	6.15	1.08		6.49	1.06		6.35	0.94	
	Seniors	16	6.50	1.11		6.22	0.94		6.32	0.91	
τ (s)	Juniors	16	5.25	0.63		5.17	0.39		5.17	0.45	
	Seniors	16	5.20	0.57		5.11	0.56		5.22	0.52	
MRT (s)	Juniors	16	11.41	1.20		11.66	1.20		11.52	1.15	
	Seniors	16	11.70	1.25		11.34	1.00		11.54	1.05	
BL (%)	Juniors	16	63.0	9.5	*	63.6	9.0	*	63.3	8.7	*
	Seniors	16	55.9	5.7		55.5	5.7		55.7	5.2	
End SmO_2_ (%)	Juniors	16	9.9	4.9		9.5	5.6		9.8	4.9	
	Seniors	16	9.6	5.0		8.6	4.7		9.0	4.6	
A_p_ (%)	Juniors	16	53.1	8.3	*	54.2	8.6	*	53.6	7.9	*
	Seniors	16	46.4	7.9		46.9	7.8		46.7	7.6	
SE_regr_ (%)	Juniors	16	1.83	0.38		1.93	0.41		1.83	0.39	
	Seniors	16	1.76	0.51		1.79	0.48		1.77	0.46	

*—significant difference between Juniors and Seniors; (*p* < 0.05; d > 0.2).

**Table 4:** Partial correlations between cyclists’ performance characteristics and SmO_2_ kinetics parameters measured during 30 s sprint effort. Power (P), 1st ventilatory threshold (VT1), 2nd ventilatory threshold (VT2), peak power (PP), oxygen uptake (VO_2_), rate of power development (RPD), time (t), power drop (PD), fatigue index (FI), lactate accumulation rate (VLa_max_).

Variable	TD (s)		τ (s)		MRT (s)		BL (%)	End SmO_2_ (%)	A_p_ (%)
P@VT1 (W/kg)	0.15		0.16		0.20		−0.14	0.05	−0.13
P@VT2 (W/kg)	0.30		0.25		0.37	*	−0.11	−0.05	−0.07
PPW (W/kg)	0.40	*	0.21		0.43	*	0.03	−0.04	0.05
VO_2_@VT1 (mL/min/kg)	0.25		0.28		0.33		0.10	0.04	0.07
VO_2_@VT2 (mL/min/kg)	0.36	*	0.24		0.41	*	−0.03	0.01	−0.04
VO_2_max (mL/min/kg)	0.47	**	0.10		0.44	*	0.17	0.15	0.06
RPD (W/s/kg)	−0.11		−0.05		0.07		0.03	−0.01	0.05
t@Pmax	−0.03		0.24		0.02		0.01	−0.09	−0.01
Pmax1s (W/kg)	−0.23		0.14		−0.13		0.00	−0.15	0.17
Pmax5s (W/kg)	−0.26		0.11		−0.17		0.05	−0.15	0.14
Pmax30s (W/kg)	−0.12		0.26		0.02		0.02	−0.18	0.13
Pmin5s (W/kg)	0.34		0.41	*	0.47	**	0.10	−0.08	0.14
PD(W/kg)	−0.46	*	−0.10		−0.42	*	0.00	−0.12	0.07
FI (%)	−0.54	**	0.01		−0.27		−0.01	−0.05	0.02
V_LAmax_ (mmol/L/s)	−0.39	*	−0.25		−0.44	*	0.03	−0.02	0.08
Phenotype (a.u.)	−0.49	**	−0.06		−0.44	*	0.09	−0.11	0.18

* *p* < 0.05, ** *p* < 0.01; controlling variable: Age Group.

## Data Availability

The data that support the findings of this study are available from the corresponding author upon reasonable request.
